# Young People’s Attitudes, Perceptions and Experiences of Social Distancing and Self-Isolation During the Second Wave of the COVID-19 Pandemic

**DOI:** 10.3389/ijph.2023.1604388

**Published:** 2023-07-03

**Authors:** Rowena Katherine Merritt, Alexandra Vastano, Jun Nakagawa, Donna Doherty-Kelly, Jayne Taylor

**Affiliations:** ^1^ Centre for Health Services Studies (CHSS), University of Kent, Canterbury, United Kingdom; ^2^ City of London Corporation & London Borough of Hackney Public Health Service, London, United Kingdom

**Keywords:** youth, COVID-19, social distancing, qualitative, self-isolation

## Abstract

**Objectives:** Social distancing and self-isolation were key parts of the UK’s strategy for reducing the spread of COVID-19. This study explored young people’s attitudes, perceptions and experiences of social distancing and social isolation during the COVID-19 pandemic.

**Methods:** Qualitative individual, family and paired-friendship interviews were conducted. All 26 participants lived or worked in East London and were aged between 20 and 39 years.

**Results:** Qualitative analysis revealed three main themes: 1) trust and breaking of the social distancing and self-isolation rules—trust in their friends to be careful and say if they are unwell; 2) own rule making—making their own household rules which made them less guilty about breaking national rules as they were adhering to rules (albeit their own); and 3) lack of clarity around self isolation and the need for practical support—confusion around length of time needed to self isolate and what self-isolation really meant.

**Conclusion:** Developing more effective and targeted communications and practical support mechanisms to encourage better adherence to social distancing and self-isolation rules among young people will be essential to prevent the spread of COVID-19.

## Introduction

On the 11th March 2020, the World Health Organisation (WHO) declared the novel coronavirus (COVID-19) outbreak a global pandemic [1]. The pandemic presents a significant challenge for public health in the UK and globally [2]. As one of the most critical elements of reducing virus transmission is public behaviour, public health teams have needed to respond with effective and appropriate interventions, policies, and messages.

There are clear preventative measures that can be taken by the public to reduce the spread of COVID-19, such as thorough and frequent handwashing, the wearing of masks in public places, social distancing and self-isolation [3]. Social distancing and self-isolation were key parts of the UK’s strategy for reducing the spread of COVID-19 [4]. However, before COVID-19 most people were probably unfamiliar with the terms “social distancing” and “self-isolation.” Social distancing in the UK banned public gatherings and focused on the recommendation of keeping a distance of 2 m apart from others [5]. In June 2020, the 2 m rule was relaxed to “one metre plus” in Scotland and England to allow people to remain one metre away if they take additional preventative measures such as wearing a face covering [6]. With self-isolation, the guidelines for England changed a total of three times from March to December of 2020. The length of the self-isolation period varied depending on whether an individual tested positive, was told they were in contact with a positive case by NHS Test and Trace, or if they lived in the same household as someone that tested positive [7, 8].

Numerous messages at national and local level were developed to inform people of the risks and the need to comply with self-isolation and social distancing. Across the UK data showed that most people adhered with the restrictions [9]. Despite this documented public support for pandemic response strategies, recent reports indicate signs of pandemic fatigue [10, 11], and that intention to practice protective measures, is greater than actual adherence. Demographic and situational factors, as well as psychological and situational factors, risk conceptualisation on COVID-19, and the public’s level of trust in the government all play a role in compliance levels [12–15]. Young people in particular are less likely to adhere to the social distancing and self-isolation rules due to a low-risk conceptualisation of COVID-19 [16].

This may imply that the public health messaging around social distancing and self-isolation are ineffective, in particular with younger audiences, and that encouraging young people to adopt such behaviours requires a more targeted approach [17], and a nuanced understanding of the social, cultural and behavioural dynamics influencing decision-making and actions. To our knowledge, there is no published qualitative evidence on young people’s perceptions and experiences of social distancing and self-isolation during COVID-19. This research study seeks to address this knowledge gap.

This study explored three main questions: i) What are the physical and psychological barriers to social distancing and self-isolation experienced by young people living in East London during the COVID-19 pandemic? ii) What are possible motivating factors which would encourage participants to social distance and self-isolate correctly? iii) What are participants’ current experiences of adherence in relation to social distancing and self-isolation?

## Methods

A mix of individual, family and friendship interviews were conducted with 26 young people (ages 20–39 years old) who lived or worked in a borough in East London (Hackney & City of London). At the time of data collection, the incidence rate of COVID-19 cases was the highest among this age group [18]. The family and friendship interviews were conducted to try and gain an understanding of the social dynamics around social distancing and self-isolation behaviours. All interviews were conducted in February 2021 either over the phone or online due to lockdown restrictions. The participants were given the option of either a phone or online interviews and were asked to select which method they felt most comfortable with. The researcher stressed that if they did not feel comfortable showing their face during the online interview, then this was completely fine also. All participants were interviewed once and interviews took between 28 min and 1.2 h, with an average length of 44 min.

All of the interviews were qualitative in nature, and this allowed the research team to understand not only what happened, for example, if social distance and self-isolate rules were adhered to, but also why they were not adhered to—what led up to them being broken, what followed, and how it made them feel.

The participants were selected using purposive sampling, meaning they were selected because they possessed knowledge that was directly related to the research questions [19, 20]. This sampling method was also used to try and increase the generalisability of the study findings. Sampling considered age, gender, location, ethnicities and life stages (Table 1) [21]. A mix of “Doers” (those who practice the behaviour) and “Non-doers” (those who do not) were recruited [22].

**TABLE 1:** Participant details, London, United Kingdom 2021.

Characteristic	n (%)
Gender	
Female	13 (50%)
Male	13 (50%)
Age range	
20–25	9 (35%)
26–30	5 (19%)
31–35	6 (23%)
36–39	2 (8%)
Over 40 years old	4 (15%)
Ethnicity	
White – British	9 (35%)
White – any other White background	10 (38%)
Black or Black British	2 (8%)
Asian or Asian British	3 (12%)
Other	2 (8%)
Occupational classification (coded using the ONS Occupation Coding Tool)	
Professional occupations	3 (12%)
Associate professional and technical occupations	4 (15%)
Administrative and secretarial occupations	1 (4%)
Skilled trade occupations	1 (4%)
Sales and customer service occupations	1 (4%)
Caring, leisure and other service occupations	5 (19%)
Elementary occupations	4 (15%)
Full-time student	1 (4%)
Homemaker	2 (8%)
Unemployed	4 (15%)

Researchers used a combination of snowball sampling and recruited through online community and volunteer advertising sites and local community groups. Due to social distancing measures, it was necessary for all recruitment to be conducted online. During recruitment, if someone expressed an interest in taking part in the study, they were emailed an information sheet which explained the study in more detail and informed them: a) why the study was being conducted, b) who was doing the study, c) how the data collected would be stored, used and analysed, and d) informing them that all interviews would be recorded and transcribed verbatim for analysis purposes. At the time of the interview, once the recoding of the interview had started, verbal consent was obtained. All of the participants were over 20 years of age and able to give consent.

An interview discussion guide was initially developed based on the findings from a literature review of articles published on self-isolation and social distancing over the past 12 months. The main topics for the interviews were: general understanding on social distancing and isolation; health impacts of social distancing and isolation for themselves and others (for example, perception of risk, perceived severity of COVID-19, etc.); barriers to adhering to the behaviours; and views on compliance within their social networks.

For this study semi-structured interviews were conducted. At the start of each interview, loosely structured, open-ended questions were asked. In order to pursue an idea or response, more detailed questions were subsequently asked, or prompts made. The wording was not standardised, as the interviewers tried to use the participant’s own vocabulary when framing supplementary questions. The guide was used as an “aide-memoire” and as a general framework for discussion, ensuring that all themes were covered with the necessary prompts but, at the same time, enabling discussions to be spontaneous, flexible, and responsive to the thoughts and opinions of those being interviewed.

To try and put the participants at ease and illicit honest responses, at the start of each interview, the researcher collecting the data (RM) stressed that there:

• Were no right or wrong answers
• She was working as an independent consultant for the local council, and no names or personal details, or transcripts would ever be shared with the council (just a summary of the key findings with anonymised quotes)
• She was only interested in their honest views and opinions, and if they agreed or disagreed with the COVID rules, or if they had followed them or not, that was fine, and that no judgment would be made either way.

### Data Analysis

Data collection and analysis followed an iterative process, whereby emergent themes from early interviews were used to add to or refine questions during subsequent interviews. All interviews were audio recorded with permission from the participants and transcribed verbatim for analysis purposes. Transcriptions were imported into NVivo (V.11.4.3, QSR) [23] and analysis followed a thematic approach to identify key themes and codes [24]. Data collection and analysis continued until saturation occurred (i.e., the point at which no new significant themes emerged).

The inductive identification of themes consisted of several stages:

• First, the initial transcripts were read several times by one researcher (RM) to generate a thorough understanding of the data. A basic “tree structure” was then formed in NVivo.
• Each transcript was read, and tentative themes allocated a code. Early in the study, the emphasis was on inclusive coding, that is, if any doubt existed about whether data described a new or existing them, a new code was created.
• Third, each transcript was coded line by line.
• Fourth, the emerging theory was used to guide further sampling, data collection and analysis which followed. New data were checked against existing theory to see if it confirmed or refuted.
• Finally, tentative categories were extended or collapsed into each other. Themes identified which were not considered central to the research questions were discarded.

### Increasing the Trustworthiness of the Study

Qualitative research is often criticised for being subject to researcher bias, and lacking reproducibility and generalisability. The following strategies were employed to enhance the trustworthiness of the study:

• Investigator triangulation

Only one researcher (RM) analysed the data. However, peer debriefings were held weekly during the analysis phases where the emerging findings were reviewed and discussed to increase the trustworthiness of the data. All authors were also involved in the design of the study and the development of the discussion guide.

• Bracketing

To reduce researcher influence, before beginning the study, the lead researcher (RM) wrote down their prior beliefs and hypotheses about the subject matter. RM also kept journal notes throughout the data collection and analysis, recording her personal feelings and biases which might have influenced her interpretation of the results.

### Interviewer’s Influence

To ensure high uniformity in the conduct of interviews, one researcher (RM) performed all the interviews. Further measures were used in an attempt to reduce potential bias caused by participants giving responses which they perceived to be desired by the researcher. The researcher emphasised:

• She did not live in the local area or know any of the local community groups supporting with the recruitment.
• The transcript of the interview would be identified only by a number.
• No identifiers would be included in any of the quotations used in publications or reports.
• The names of anybody mentioned, along with any other identifying information, would be removed from the transcripts before analysis.
• Audit trail

The computer program NVivo was used throughout the data gathering and analysis. This permitted an extensive audit trail, formed by the memos attached to codes, interview transcripts, and field notes. This audit trail provides a record of the development of the project from the start to finish and, theoretically, this would allow someone not connected with the study to review the primary documents and coding schemes to assess the findings, interpretations, and conclusions are supported.

## Results

Analysis revealed three broad themes around [1]: trust and how this affected adherence to the social distancing and self-isolation rules [2]; rule making; and [3] lack of clarity around social isolation and the need for practical support.

### Trust in Others in Relation to Adherence of the COVID Rules

Whilst all of the participants were aware of the rules around social distancing, they were frequently disregarded by most of the participants. All of the participants stated they would wear a face mask if they needed to go to a shop or travel on public transport. This made them feel “more comfortable” and “less nervous.” They felt as if they needed to wear a mask and practice good hand hygiene when they were in a public space as they could “not trust” other people to practice the same safety precautions. However, there was a vastly different perception of risk if they were meeting up with friends or family members who they trusted.

“If you go to a shop, it is in everyone’s mind. But if you go to a park say, a public place, there is something about knowing the people you are with, even if you don’t live with them. I was hanging out with friends and then someone invites another girl and before you know it, it is a big group. But your comfort level is… you know some of them…. and then you trust them.”

“I make a point not to break the rules as I think maybe they live with a vulnerable person. But I have broken the rules. When I was meeting with a friend who lives with her boyfriend, and I think I don’t see anyone else, and they don’t see anyone else. So, it is like you are being careful and you justify it in your head.”

“We met outside as we are not the same flat mate, and I had a really good feeling and I said, how many people have you seen recently, and he said, ‘I’m working from home’, and I told him, ‘I had a COVID test’ and so I say, ‘OK I’m going to kiss him’. I don’t know… it was like a…. but it is weird, but I trust him as I saw him and spoke to him a lot…”

Even when meeting with friends, observing their hygiene practices during the pandemic was interlinked with trust. As one participant explained: “I trust this guy [man whom she is dating] as he is very clean and uses hand sanitizer, and you notice these things. And there is another friend of mine, and he had an old mask, and I was ok, ‘oh OK, I don’t want to see him’. I can’t trust him.”

The fear of the virus and their perception of risk was dynamic; it constantly changed based on reported deaths, local infection rates, and word-of-mouth - predominantly from other friends and family members who had themselves contracted COVID-19 or who shared news articles. This perception of risk influenced social distancing adherence.

“We are by and large sticking to the rules, but… We go through waves, sometimes I am like, ‘oh sod it’ [break the rules]. But when I have listened to a news bulletin….”

“I thought it was great, in the summer as you could go for a walk and your mental health was so much better, but my parents would freak me out by sending me these articles it is in the air.”

The participants often justified their lack of social distancing by saying they only met up with people “in their bubble.” However, as one participant explained, they could have more than one support bubble, but by using the term “support bubble” it felt as if they could justify their lack of social distancing more comfortably: “We hug and no social distancing as we justify it in our heads that we are each others support bubbles. But in reality, I have been seeing [girlfriends name], but as we have labelled it, it is OK.”

Although the rules around social distancing were broken by nearly all of the participants at one time or another, they did not take the decision lightly and on the whole they tried to adhere to the rules. However, by the time the UK entered their third lockdown, they simply felt the need to have “human contact.” They felt alone and isolated and for most people, meeting up with friends was seen as a last resort and something they needed to do as they had reached “breaking point.”

“I think I have already been fearful of it, but I think my willingness to take on the risk, if I am at the end of my rope and I need socialisation I do decide I am willing to take the risk as I need it to feel normal again. Then you see people you can’t see, you feel better, but then all the doubt comes afterwards until the next time you reach your breaking point.”

A few of the participants explained that they often felt guilty for not adhering to the social distancing rules.

“Every time after I have knowingly broken a rule, I always the next day I always feel ill, out of guilt. I think – I deserve this.”

However, most people would feel “revitalised” after meeting up with friends.

“It is very lonely. We were ready to start our London life, but I haven’t met anyone new in a year. One friend had a very illegal birthday party which I did go to and I met some new people, and it was so revitalising.”

“You make a personal choice on whether or not you hug each other. And then, we all just decided to hug, and you go and say do you want to hug but you always end up hugging. We shouldn’t be doing it, but it feels so good even if it is just for a second.”

### How People Developed Their Own Rules During COVID Lockdowns

Most households appeared to have their own rules around social distancing and self-isolation. For example, one household allowed the daughter to see her boyfriend twice a week, if they both agreed to “be careful” the rest of the time. Another interview with flatmates revealed that their rule was each flat mate could have a set amount of people (maximum 3 people) who could come over during the lockdown period. As there were four people living in the flat share, this resulted in 15 people coming and going in that one household. One of the participants interviewed felt frustrated by her flatmate’s attitudes. She believed that by having your own rules it meant people broke the national rules: “They bend the rules by making their own rules for the house and by having rules, it makes them feel as they still have rules and therefore are following the rules…. but it is not the rules that help us.”

However, by having their own rules, most participants felt that they were “being good” and were adhering to the rules (even though these were not the national government rules).

### Lack of Clarity Around Social Isolation and the Need for Practical Support

Whilst participants were often happy to talk about social distancing and were honest about the times they had (in their own view) “broken the rules”, they were more hesitant and reserved when talking about self-isolation. Some also changed their narrative through the course of the interview, first stating that they had self-isolated to then admitting they did not “totally” self-isolate.

*Interviewer: “*So at the start you mentioned you self-isolated, can you tell me a bit more about that?

*Participant:* Yes… I…. I might as well be honest with you as I guess there is no use in me not being…. I probably should have but I didn’t totally……”

In relation to self-isolation, nearly all of the participants were unclear of when you should self-isolate, for how long, and what was and was not allowed. For example, they questioned—was it only if they have COVID-19 themselves? Or if they have come in contact with someone who has COVID-19? There was also confusion around the length of time they needed to self-isolate with some people believing it was 14 days, whilst others stating ten or 7 days, or until they returned a negative test result.

This lack of awareness often caused confusion and ended up with participants making up their own rules and interpreting the term “self-isolation” in their own way. For example, a few stated that you can still go to public parks.

“Umm…. self isolation means no contact with anyone else. When I had to self-isolate upon my return from America, my family and I went for walks and to run around. But we didn’t touch anything and wore masks. So self isolation means not touching anything or coughing on anyone, no contact with anyone. But it doesn’t mean staying in your house.”

Despite the confusion around self-isolation and the perceived difficulty, the participants were keen to stress that they would self-isolate if they tested positive. They were conscientious and reported that they would feel guilty if they spread the virus. A couple even talked about the shame and the stigma they would feel if they had unwittingly passed on the virus to other people.

“I would never think that of someone else but if I was to get it, I would feel a stigma. Especially having to contact everyone and say… but I feel if I got it, I’d think, I’d have to tell everyone at work and I’d be that person, that person who wasn’t being careful. But I’d never dream of stigmatising someone else.”

At the same time, many of the participants also emphasised that self-isolating is difficult. The young people explained how they “*dreaded it*” and did not know “*how they would cope*”.

“If someone contacted me and they said they had tested positive, I would get a test and see if I had got it. But I don’t know how long it takes to take systems. But I would see but it is a real ball ache so if I can avoid self isolating, I will do. I don’t want to sit in my flat for 14 days.”

“And then I was worried, if I get it and I am going to get sick, who is going to take care of me? My flat mate? I don’t want them. I don’t have friends here so what am I going to do?”

## Discussion

### Rule Setting During COVID Lockdowns

Unlike national survey data that indicate a high compliance with social distancing and self-isolation rules [25], participants in this study often described partaking in activities that were not always in adherence with the government’s social distancing and self-isolation rules. However, whilst there are numerous news reports suggesting that people wilfully break the rules with little consideration for others or regard for the consequences, this research shows that most of the young people interviewed only failed to adhere to the rules when they felt at “breaking point.” This study also highlighted that the lack of adherence was not always high-risk and many of the participants considered the risks and tried to minimise them. This “breaking point,” often led to them developing their own rules.

By setting their own rules, partial adherence to the government guidelines became more common, and by having their own rules, they felt they were adhering, at some level, to the guidelines.

### Understanding Who Young People Trusted and How Trust Influenced Their Adherence to the Rules

The participants would also emphasise that they were not attending house parties, instead they are meeting up with a small group of “trusted” friends. For some participants, their group of trusted friends would be very small, whereas for others it would be much larger. However, this finding would still suggest that the media’s focus on the house parties is misrepresenting the problem of adherence, and also undermines the way young people respond to the issue by making them feel as if their own actions are more justified.

As well as confusion over length of time, this study found that the young people had concerns around how they could practically adhere to the rules. This was particularly prominent for the participants living in houses in multiple occupation (flat shares), and whom did not have personal support networks locally. Although services are available locally, the messages around these support services appear to not be reaching all audiences. This finding also linked back into their perception of “trust”, as they did not always trust their flatmates to adhere to the rules.

### Lack of Clarity in Relation to the Changing Rules

Overall, this study found that there was a good understanding of the need to adhere to the self-isolation guidelines. However, there was confusion around the length of time someone would need to self-isolate for if they displayed symptoms or had come into contact with someone who had tested positive for COVID-19. Since March 2020, the guidelines have changed three times, and the time needed to self-isolate has been reduced from 14 to 10 days. The four Chief Medical Officers in the UK believed that the reduction in days would help with compliance rates [26]. Government guidance on how to adhere to self-isolation as well as for how long, often left room for ambiguity, and communications around this appear to assume that the general public had some prior knowledge and therefore did not always specify exactly what was needed to properly self-isolate and book a test [27]. This study suggests that the changing guidelines and their lack of clarity have created confusion and that the messages around the isolation periods are not reaching the younger audiences effectively.

### Relevance to Existing Literature

Our findings on COVID-19 social distancing and self-isolation support the findings from other studies and literature reviews. For instance, we found that lack of clarity in communications and the practicalities of adherence were all major barriers [11, 13, 15, 24]. In much of the research, lower adherence was associated with being male, being younger in age, having a dependent child in the household, being of lower socio-economic grade, and being less informed about COVID-19 and guidance to prevent the spread of the virus (e.g., not being able to identify key symptoms of COVID-19, not knowing government guidance if you were to develop symptoms of COVID-19, and disagreeing that someone can spread COVID-19 even if they are asymptomatic) [11–15, 26–30]. However, to date, research has not focused on investigating, in detail, what people are doing, why, and how safe it is. Previous research has also not focused on participant knowledge of the practical and emotional support available to help them self-isolate. This research has highlighted that there are varying degrees of adherence as well as subjective rule interpretation and own rule-making, meaning that different forms of messaging is needed to support individuals in protecting themselves, their households, and their communities from COVID-19.

### Implications for Policy and Practice

This study suggests that there needs to be a more insightful understanding and evaluation of compliance with social distancing and self-isolation guidelines. In practice, this could mean rephrasing existing national survey questions that only assess whether the respondent has self-isolated or not [31]. A more nuanced phrasing could capture partial adherence and help policymakers and researchers identify gaps in the public’s understanding of the guidelines.

Our findings also suggest the need for more targeted communications catered to a younger audience instead of a “one-size-fits-all” approach. With the government’s roadmap out of lockdown, future research in this area will be key to reduce the likelihood of young people reaching the “breaking point” too early, and for communications to work closely with local services to ensure that people who are close to “breaking point” are signposted to the available support services, likely to be digital. Future policy should also seek to provide more support for self-isolation targeting young people who are more likely to live in shared accommodations where full adherence may be challenging or impossible. Other studies have proposed that better financial support may help improve adherence to self-isolation compliance [32, 33], however it is also the practical and emotional support resulting from the lack of social networks which is also vital.

### Limitations

The sample size for this study was small (although data saturation was reached) and data was only collected in one part of London. Data was also collected for this study during the third UK lockdown when there were strict restrictions in place, and before the “road map” out of lockdown had been published. Although it provides a good snapshot into that moment in time, as the lockdown measures ease, changes in perceptions, attitudes and behaviours can rapidly change, and transferring the findings to any future lockdowns should be made with caution.

Due to the restrictions at the time of data collection, all sampling and interviews were all done online or over the phone. Therefore, only people who used the internet and were happy to be interviewed virtually were able to take part, which may have resulted in a biased sample.

Although efforts were made to reassure participants around confidentiality and non-judgment during the interview process, some participants may have felt nervous to express their honest opinion if it went against the government rules.

### Conclusion

Since many young people will be among the last to be offered the COVID-19 vaccine, finding effective communication strategies and support mechanisms to encourage better adherence to social distancing and self-isolation rules will be essential to prevent the spread of COVID-19. Any campaign messages and strategies should consider the findings of this study, to help young people think through their understanding of “trust,” possibly by highlighting just how many people their trusted friends might encounter (for example, takeaway delivery person, postman, etc.). To support compliance with social isolation, messages could also use positive reinforcement or comedy to imitate real life situations acknowledging the issues of self-isolation in a flat share but promote where young people can get support.
